# Habitual patellar dislocation exhibits less severe rotational deformities but poorer trochlear development and higher incidence of patella baja compared with recurrent patellar dislocation in skeletally mature patients

**DOI:** 10.1002/ksa.12392

**Published:** 2024-08-09

**Authors:** Zhijun Zhang, Zheng Feng, Menglinqian Di, Daofeng Wang, Tong Zheng, Hui Zhang

**Affiliations:** ^1^ Sports Medicine Service, Beijing jishuitan hospital Capital Medical University Beijing China; ^2^ Beijing Research Institute of Traumatology and Orthopaedics Beijing China

**Keywords:** habitual patellar dislocation, patella baja, recurrent patellar dislocation, skeletally mature patients

## Abstract

**Purpose:**

This study compared the radiological characteristics between habitual and recurrent patellar dislocation in skeletally mature patients.

**Methods:**

From 2017 to 2019, 77 skeletally mature patients with habitual patellar dislocation were surgically treated at a single institution and reviewed retrospectively. A total of 55 knees from these patients were included in the habitual patellar dislocation group. During the same period, 55 knees with recurrent patellar dislocation were randomly selected from 242 patients and included in the recurrent patellar dislocation group. Various bony deformities were measured and compared between the two groups. Additionally, a subgroup analysis was conducted among patients with habitual patellar dislocation, comparing those with and without an ‘invisible patella’ observed on true lateral views with 30° of knee flexion.

**Results:**

The femoral anteversion angle (21.8° vs. 26.3°, *p* = 0.041), tibiofemoral rotation angle (9.7° vs. 12.4°, *p* = 0.042) and external tibial rotation angle (24.3° vs. 29.6°, *p* = 0.001) in the habitual patellar dislocation group were significantly lower than those in the recurrent patellar dislocation group. 54% of knees in the habitual patellar dislocation group had a patella baja, and this was in sharp contrast to the recurrent patellar dislocation group in which none of the knees had a patella baja. 49.1% of knees in the habitual patellar dislocation group showed ‘invisible patella’ at 30° of knee flexion, and knees with ‘invisible patella’ had significantly higher tibial tubercle‐trochlear groove (TT‐TG) distance (30.4 vs. 19.8, *p* < 0.001) and tibiofemoral rotation angle (13.2° vs. 6.4°, *p* < 0.001) than knees with a visible patella.

**Conclusions:**

A distinct difference in bony anatomical features was observed between habitual and recurrent patellar dislocation in skeletally mature patients. Habitual patellar dislocation exhibited less severe rotational deformities of the lower extremity but showed poorer trochlear and patellar development, a larger TT‐TG distance and a higher incidence of patella baja compared with recurrent patellar dislocation.

**Level of Evidence:**

Level III.

**Abbreviations**
CDI: Caton–Deschamps index
mFTA: mechanical femoral‐tibial angle
TT‐TG: tibial tubercle‐trochlear groove

## INTRODUCTION

Habitual patellar dislocation represents the most severe form of patellar instability, characterised by the patella dislocating every time the knee is flexed [2]. Patients typically experience an abnormal appearance of the knee and sensations of instability in the early stages of life [15, 20]. Although these children can learn to compensate, the instability frequently gets worse with growth [21]. In contrast, recurrent patellar dislocation is a more typical pattern of patellar instability, involving repeated dislocations following an initial episode often triggered by minor trauma.

Several factors contribute to the pathogenesis of habitual patellar dislocation, with the most significant pathological factors being the shortened quadriceps mechanism and contracture of the lateral soft tissues [2, 12, 13, 26]. Various proximal and distal realignment procedures have been described in the literature for treating habitual patellar dislocation in children [2]. Among these procedures, a treatment algorithm emphasising quadriceps lengthening is essential for improving patellar tracking during deep knee flexion [12, 26].

Surgery for habitual patellar dislocation is typically recommended without delay due to the risk of painful arthrosis because the trochlea has a greater potential for remodelling when patellar stabilisation occurs at a younger age [18]. However, some children with habitual patellar dislocation may tolerate their condition reasonably well without pain or apprehension. It is not uncommon for these patients to delay surgical intervention until adulthood, seeking treatment primarily for cosmetic abnormalities or functional issues with the knee [6, 20, 21, 22].

In skeletally mature patients with habitual patellar dislocation, the condition often presents with more severe quadriceps contracture and complex bony deformities, posing significant challenges for treatment [21]. Surgical approaches in these cases may include bony procedures aimed at realigning and stabilising the patellofemoral joint, which differs from the predominantly soft tissue surgeries performed in children [2, 10, 14].

A thorough understanding of the bony deformities present in skeletally mature patients with habitual patellar dislocation can guide surgeons in performing targeted bony correction surgeries aimed at improving patellofemoral alignment. Despite this, there is a paucity of literature regarding the radiographic features of habitual patellar dislocation in skeletally mature patients [20, 21]. Therefore, the objective of this study is to assess radiographic parameters in a relatively large cohort of skeletally mature patients diagnosed with habitual patellar dislocation and to compare these findings with those of patients experiencing recurrent patellar dislocation. The hypothesis posited is that habitual patellar dislocation may exhibit distinct radiographic parameters when compared with recurrent patellar dislocation.

## MATERIALS AND METHODS

This study was conducted in accordance with the approval of the ethics board of Beijing Jishuitan Hospital and adhered to all patient consent requirements (approval number 20231011). At Beijing Jishuitan Hospital, skeletally immature patients with habitual or recurrent patellar dislocation were managed by the paediatric orthopaedics department, whereas surgical treatment for patients older than 14 years was provided by the sports medicine department. Consequently, only skeletally mature patients were included in this analysis.

From 2017 to 2019, a total of 77 consecutive skeletally mature patients (representing 83 affected knees with closed growth plates) diagnosed with habitual patellar dislocation underwent surgical treatment and were included in this retrospective review. Patients meeting any of the following exclusion criteria were not included: (1) cases requiring revision surgery, and (2) patients lacking preoperative radiological data.

Twenty‐six revision cases were excluded from the study. Their primary surgeries included: 17 cases of lateral release and medial plication, two cases of lateral release combined with lengthening and patellar tendon hemi‐transfer and one case of isolated medial patellofemoral ligament reconstruction. The primary surgeries of six cases remained unknown.

**Figure 1:**
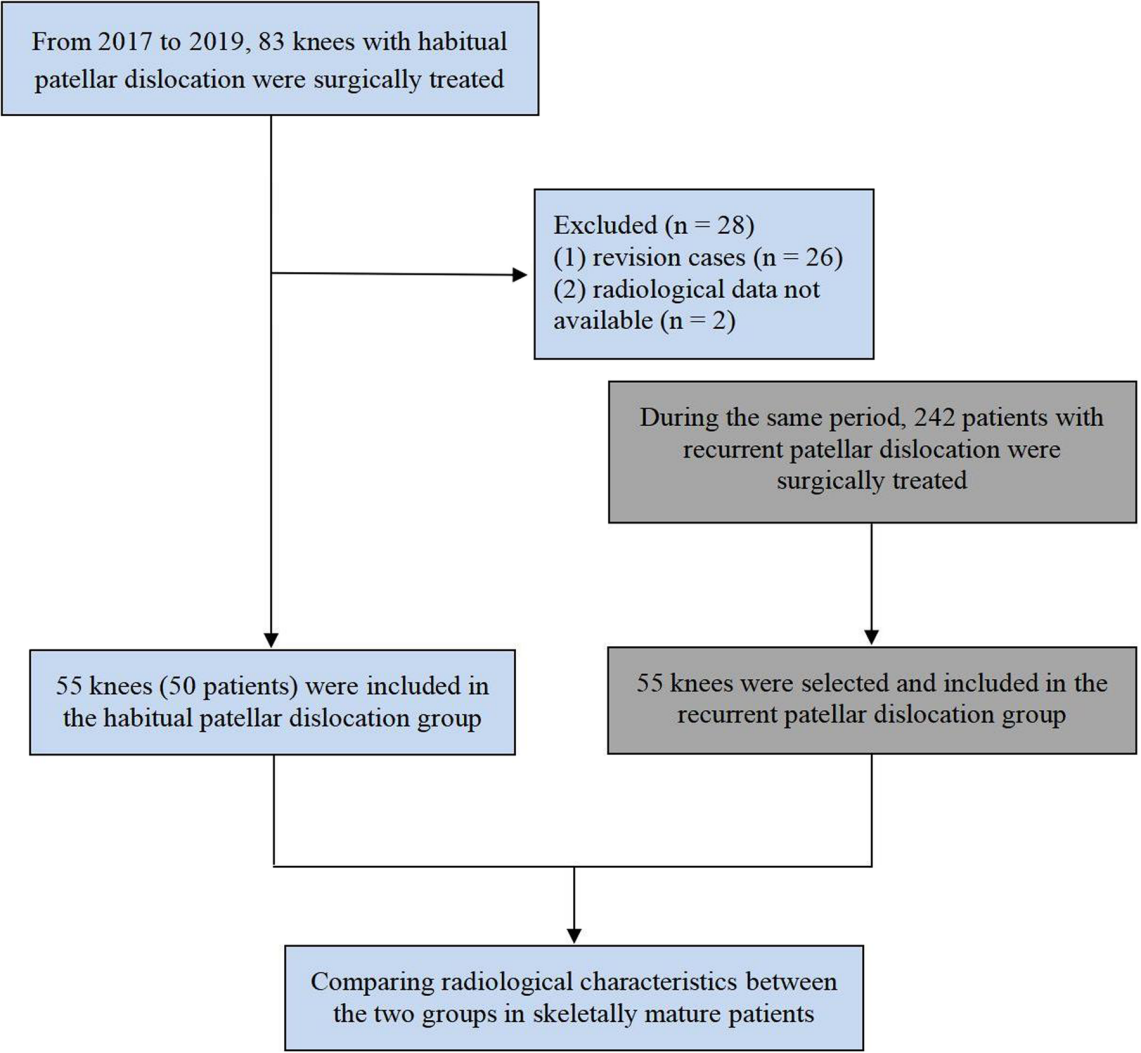
Flow chart of patient selection.

Finally, a total of 55 knees (50 patients) that underwent surgery were included in the habitual patellar dislocation group. Concurrently, during the same timeframe, 242 patients with recurrent patellar dislocation underwent surgical treatment. The recurrent patellar dislocation group was selected at a 1:1 ratio to the habitual patellar dislocation group, matching criteria included age within 5 years, body mass index within 2 kg/m², and similar primary surgical interventions (see Figure 1).

### Clinical assessment

The following information was systematically collected from the medical records: sex, age at the time of surgery, affected side (left or right), duration of symptoms and whether the condition was unilateral or bilateral.

Before surgery, dynamic evaluation of patellar tracking throughout the full range of motion was conducted for patients diagnosed with habitual patellar dislocation. In all cases, the patella dislocated upon knee flexion. A specific measurement termed the ‘dislocation angle’ was recorded, which corresponds to the knee angle at which the patella begins to dislocate. This angle serves as an approximate indicator of the severity of quadriceps contracture [21]. During knee extension, spontaneous patellar reduction occurred either fully or partially, resulting in significantly improved patellofemoral congruence.

### Radiologic measurement

**Figure 2:**
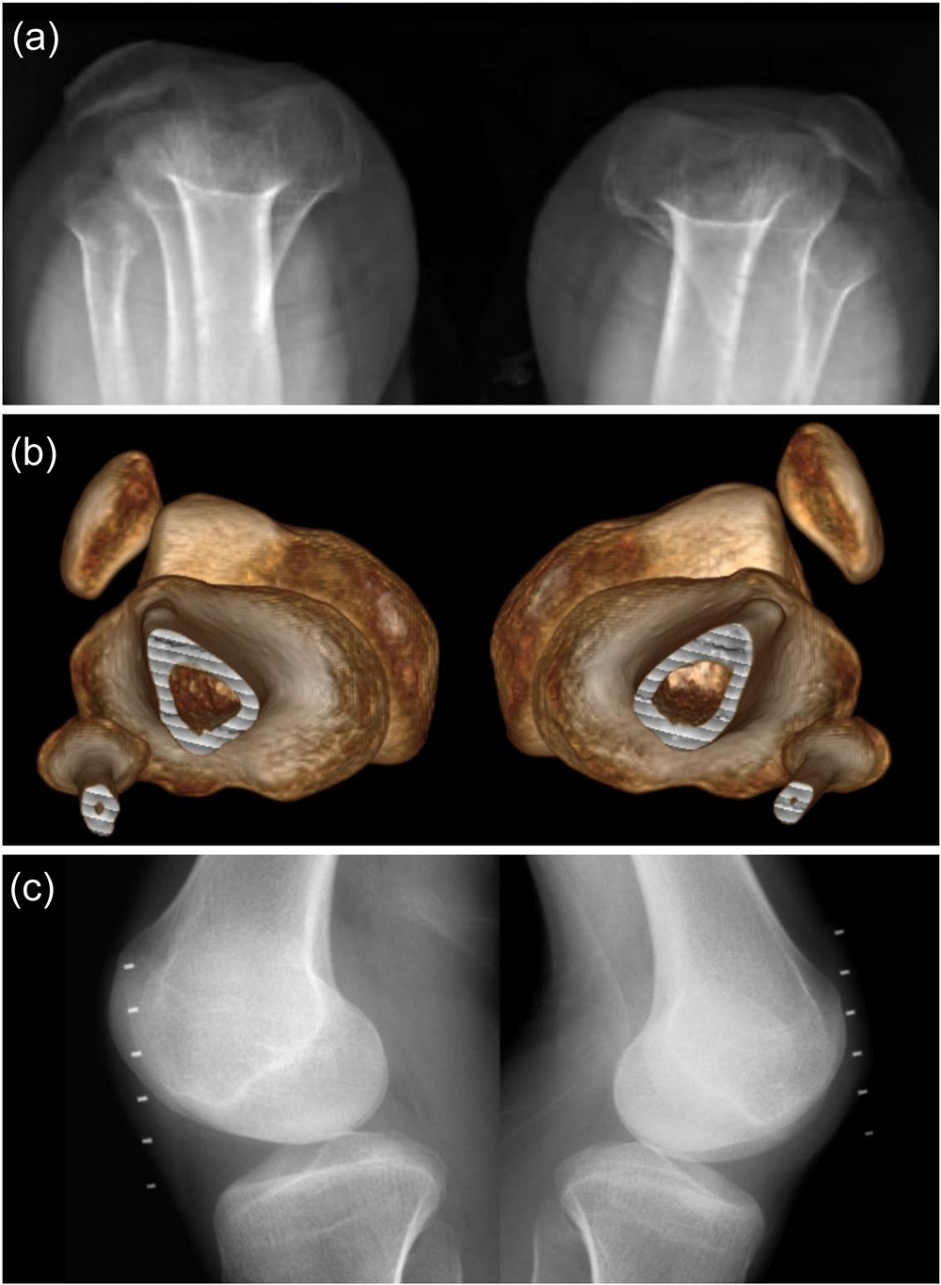
Various radiological findings associated with habitual patellar dislocation: (a) Axial views of the patellofemoral joint at maximum knee flexion angle, showing lateral dislocation of the patella. (b) Bilateral severe habitual patellar dislocation depicted in a three‐dimensional computed tomography scan, demonstrating lateral dislocation of the patella on both knees when extended. (c) ‘Invisible patella’ phenomenon, where the patella is already laterally dislocated at 30° of knee flexion. This condition prevents accurate measurement of the Caton–Deschamps index due to the patella's displaced position.

All patients included in the study underwent preoperative computed tomography (CT) scans and X‐rays, which included routine anteroposterior views, true lateral views at 30° of knee flexion, and axial views of the patellofemoral joint at both 30° and at maximum knee flexion angles (see Figure 2).

The Caton–Deschamps index (CDI) was utilised to assess patellar height. Patella alta was defined as CDI ≥ 1.2, whereas patella baja was defined as CDI ≤ 0.8 [19]. In cases where the patella was already laterally dislocated at 30° of knee flexion among patients with habitual patellar dislocation, the CDI measurement could not be obtained [13]. This condition was classified as ‘invisible patella’ (see Figure 2c).

Trochlear dysplasia was detected on the true lateral view of the knee and was classified according to the Dejour classification system [11]. The morphology of the patella was graded using the Wiberg classifications [17]. The mechanical femoral–tibial angle (mFTA) was measured on the whole‐leg standing anteroposterior radiograph to detect the presence of severe valgus deformity (mFTA ≥ 8°). Tibial tubercle‐trochlear groove (TT‐TG) distance was measured on CT [5].

**Figure 3:**
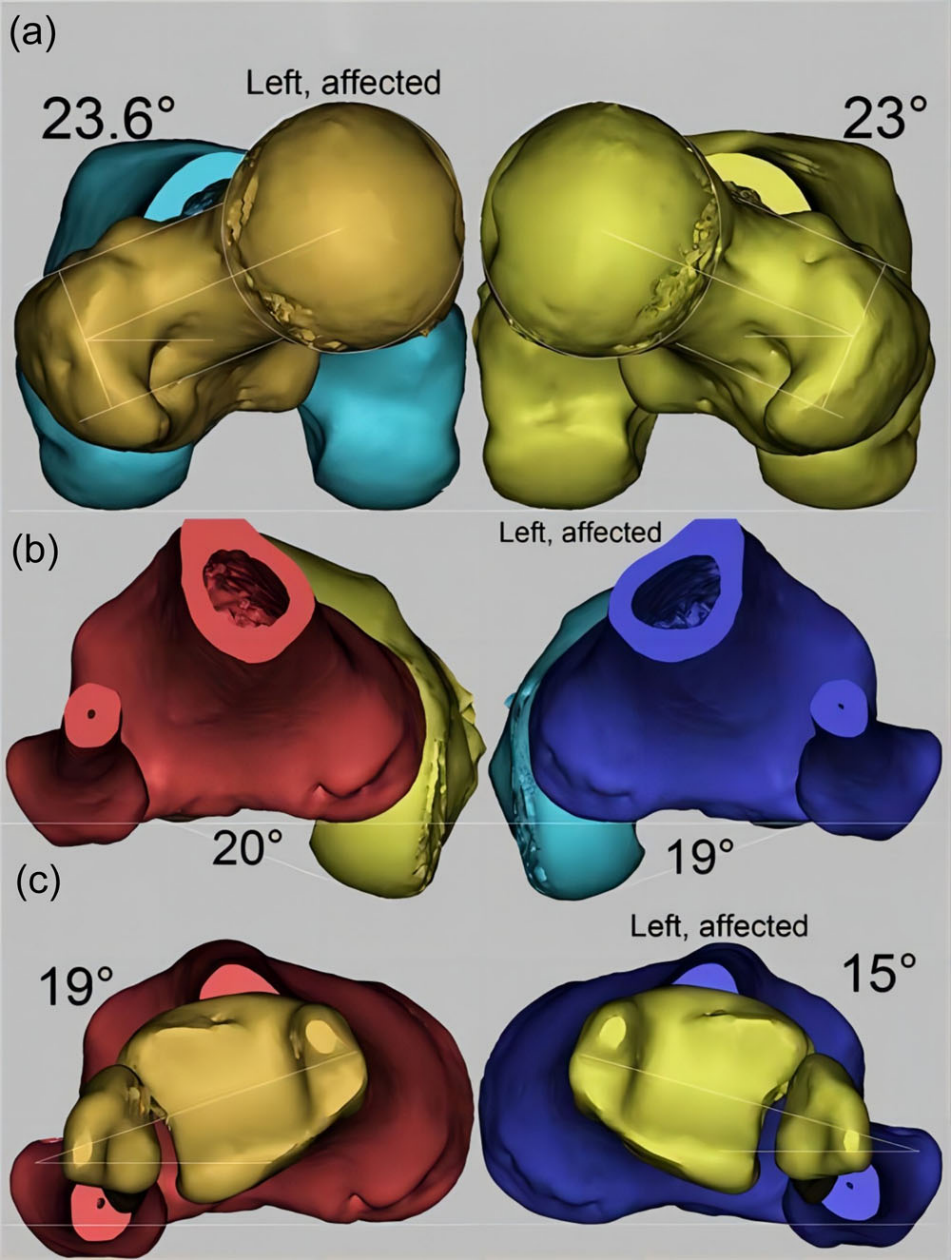
Measurement of rotational deformities of the lower extremity. (a) The femoral anteversion angle is defined as the angle formed between the axis of the femoral neck and the posterior condylar line of the distal femur. (b) The tibiofemoral rotation angle is measured by comparing the angle between the posterior condylar line and the posterior tibial condylar line. (c) The external tibial rotation angle is assessed by measuring the rotational angle of the distal tibia relative to the proximal tibia.

DICOM data obtained from hip–knee–ankle CT scans were processed using Mimics Research 20.0 software (Materialise) to create three‐dimensional models, following a methodology detailed in previous studies (see Figure 3) [23]. Several rotational parameters of the lower extremity were measured using these models.

#### Femoral anteversion angle

This angle (normal value: 17.0°) is defined as the angle formed between the axis of the femoral head–neck and the posterior condylar line of the distal femur.

#### Tibiofemoral rotation angle

This angle (normal value: 5.4°) is determined by comparing the angle between the posterior condylar line of the femur and the posterior tibial condylar line. A positive angle indicates external rotation of the proximal tibia relative to the distal femur [28].

#### External tibial rotation angle

This angle assesses the rotational alignment of the distal tibia relative to the proximal tibia [23].

All measurements in the study were conducted independently by two surgeons. The interobserver agreement was assessed and found to be as follows: 0.6° for the femoral anteversion angle, 0.7° for the tibiofemoral rotation angle, 0.5° for the external tibial rotation angle, 0.6° for mFTA, 0.1 for the CDI and 0.3 mm for the TT‐TG distance. Therefore, the measurement accuracy for all parameters was recorded to one decimal place.

### Statistical analyses

Statistical analyses were conducted using SPSS 20.0 software (IBM). Categorical variables were compared using Pearson's *χ* ^2^ test or Fisher's exact test, depending on the expected cell counts. Continuous variables were compared using either student's *t* test or Mann–Whitney *U* test, depending on the normality of data distribution. To assess the statistical power of the study, a post hoc power analysis was performed using G*Power 3.1.9.7 software (Franz Paul). The effect size used for the power analysis was determined to be 0.72, based on the TT‐TG distance measurement. With this effect size, a significance level (*α*) of 0.05 and a calculated power of 0.96 were achieved.

## RESULTS

### Baseline characteristics

There were significant differences in the population characteristics between the habitual patellar dislocation group and recurrent patellar dislocation group: The habitual patellar dislocation group had a higher proportion of male patients (43.6% vs. 12.7%, *p* < 0.001), and 21 patients in the habitual patellar dislocation group had bilateral involvement, which was significantly higher than the recurrent patellar dislocation group (38.1% vs. 18.2%, *p* = 0.02).

In the habitual patellar dislocation group, the patella remained unreduced when the knee was fully extended in 11 out of 55 knees, constituting 20% of the cases. Among the remaining 44 knees, where the patella did reduce, the mean dislocation angle at which the patella dislocated was 30 ± 32°, ranging from 0° to 100°.

### Radiological results

#### Rotational deformities of the lower extremity

The femoral anteversion angle (21.8 ± 10.5° vs. 26.3 ± 12.2°, *p* = 0.041), tibiofemoral rotation angle (9.7 ± 7.8° vs. 12.4 ± 5.7°, *p* = 0.042) and external tibial rotation angle (24.3 ± 9.6° vs. 29.6 ± 7.4°, *p* = 0.001) in the habitual patellar dislocation group were significantly lower than that in the recurrent patellar dislocation group (Table 1).

**Table 1:** Basic information for each group of participants.^a^

	Recurrent patellar dislocation group (*n* = 55)	Habitual patellar dislocation group (*n* = 55)	*p* Value
Age (year)	21.7 ± 8.1	24.5 ± 9.1	n.s.
Sex (female/male) (*n*)	48/7	31/24	**<0.001**
Affected side (L/R) (*n*)	29/26	28/27	n.s.
Bilateral affected, *n* (%)	10 (18.2)	21 (38.2)	**<0.001**
Duration of symptoms, year	6 ± 5	12 ± 8	**<0.001**
Severe valgus, *n* (%)	0 (0)	5 (9%)	n.s.
Dejour type, *n* (%)			**<0.001**
Normal	4 (7)	0 (0)	
Type A	22 (40)	8 (15)	
Type B	20 (36)	9 (16)	
Type C	2 (4)	34 (62)	
Type D	7 (13)	4 (7)	
Wiberg classification			**<0.001**
Type 1	0 (0)	8 (14.5)	
Type 2	6 (10.9)	44 (80.0)	
Type 3	49 (89.1)	3 (5.5)	
Patellar height			
CDI	1.2 ± 0.2	0.8 ± 0.2^b^	**<0.001**
Patella alta, *n* (%)	26 (47)	1 (4)	**<0.001**
Patella baja, *n* (%)	0 (0)	15 (54)	**<0.001**
TT‐TG (mm)	20.3 ± 4.0	25.0 ± 8.3	**<0.001**
Femoral anteversion (°)	26.3 ± 12.2	21.8 ± 10.5	**0.041**
Tibiofemoral rotation (°)	12.4 ± 5.7	9.7 ± 7.8	**0.042**
External tibial rotation (°)	29.6 ± 7.4	24.3 ± 9.6	**0.001**

Abbreviations: CDI, Caton−Deschamps index; L/R, left/right; TT‐TG, tibial tuberosity‐trochlear groove.

^a^ Bolded values indicate statistical significance (*p* < 0.05).

#### Patellar height

CDI was not reported in 27 (49.1%) knees with ‘invisible patella’ at 30° of knee flexion in the habitual patellar dislocation group. For the remaining 28 knees (50.9%), the mean CDI was significantly lower than in the recurrent patellar dislocation group (0.8 ± 0.2 (range, 0.36−1.23) versus 1.2 ± 0.2 (range, 0.81−1.59), *p* < 0.001). 47% of the knees (26/55) in the recurrent patellar dislocation group had a patella alta, however, 54% of knees (15/28) in the habitual patellar dislocation group had a patella baja, and this was in sharp contrast to the recurrent patellar dislocation group in which none of the knees had a patella baja (0/55) (Table 1).

#### Trochlear and patellar dysplasia

The proportion of severe trochlear dysplasia (Dejour types B−D) was significantly higher in the habitual patellar dislocation group than in the recurrent patellar dislocation group (85.5% vs. 52.7%, *p* < 0.001). In the recurrent patellar dislocation group, type A trochlear dysplasia was the most common type (40%) and type C was the least common type (4%), however, 62% of knees in the habitual patellar dislocation group had a type C trochlear dysplasia and significant differences were observed in the distribution of Dejour and Wiberg classification between the two groups, as shown in Table 1.

#### Other measurements

TT‐TG distance was significantly larger in the habitual patellar dislocation group than in the recurrent patellar dislocation group (25.0 vs. 20.3, *p* < 0.001). The habitual patellar dislocation group had a higher prevalence of severe valgus deformity, but this difference was not statistically significant (9% vs. 0%, n.s.) (Table 1).

#### Comparison of habitual patellar dislocation with and without an ‘invisible patella’

The results of the comparison between habitual patellar dislocation with and without an ‘invisible patella’ are shown in Table 2. Knees with an ‘invisible patella’ had significantly higher TT‐TG distance (30.4 vs. 19.8, *p* < 0.001) and higher tibiofemoral rotation angle (13.2° vs. 6.4°, *p* < 0.001) than knees with a visible patella (Table 2). No significant difference was found in terms of other variables.

**Table 2:** Comparison between habitual patellar dislocation with and without ‘Invisible patella’.^a^

	Visible patella (*n* = 28)	Invisible patella (*n* = 27)	*p*
Age (years)	26.0 ± 11.4	23.0 ± 5.6	n.s.
TT‐TG (mm)	19.8 ± 5.9	30.4 ± 7.0	**<0.001**
Femoral anteversion (°)	21.6 ± 10.8	22.0 ± 10.4	n.s.
Tibiofemoral rotation (°)	6.4 ± 5.3	13.2 ± 8.4	**<0.001**
External tibial rotation (°)	25.2 ± 8.6	23.2 ± 10.6	n.s.
CDI	0.8 ± 0.2	/	/
Severe valgus, *n* (%)	1 (3.6)	4 (14.8)	n.s.
Severe trochlear dysplasia, *n* (%)	22 (78.6)	25 (92.6)	n.s.
Full range of motion, *n* (%)	28 (100)	27 (100)	n.s.

Abbreviations: CDI, Caton−Deschamps index; TT‐TG, tibial tuberosity‐trochlear groove.

^a^ Bolded values indicate statistical significance (*p* < 0.05).

## DISCUSSION

The primary finding of this study reveals significant differences in bony anatomical features between habitual patellar dislocation and recurrent patellar dislocation in skeletally mature patients. This study, which includes the largest series analysed to date comprising 55 cases of habitual patellar dislocation, provides a detailed anatomical characterisation of this condition in skeletally mature patients. Our findings indicate that habitual patellar dislocation is associated with less severe rotational deformities of the lower extremity compared with the recurrent patellar dislocation. However, patients with habitual patellar dislocation demonstrate poorer trochlear and patellar development, a larger tibial TT‐TG distance and a higher incidence of patella baja when compared with recurrent patellar dislocation.

Although some children with habitual patellar dislocation may tolerate the condition without experiencing pain or instability, it is crucial to recognise and manage this condition promptly. Delayed treatment can lead to increased patellofemoral pain and weakness, potentially necessitating more extensive surgical interventions [21, 26]. Persistent abnormal patellar tracking in habitual patellar dislocation creates an unfavourable mechanical environment during lower extremity development, resulting in uneven load distribution that complicates the disease progression. Therefore, managing habitual patellar dislocation in children versus skeletally mature patients involves distinct considerations beyond age alone. The substantial differences between these patient groups necessitate unique treatment approaches tailored to each population. This study provides a comprehensive evaluation of the bony deformities observed in skeletally mature patients with habitual patellar dislocation, comparing them directly with those with recurrent patellar dislocation. These findings offer valuable anatomical insights that can guide surgeons in determining appropriate bony procedures when addressing cases where the patella remains unreduced at full knee flexion despite thorough soft tissue surgery in skeletally mature patients with habitual patellar dislocation.

The relationship between torsional deformities of the lower extremity and recurrent patellar dislocation is currently a prominent area of research [1, 4, 8, 24, 25, 27, 28, 29, 30]. However, there is limited literature on rotational deformities among patients with habitual patellar dislocation, particularly, in skeletally mature individuals. Hart et al. investigated rotational deformities in adults with habitual patellar dislocation but did not specify the degree of these deformities. They performed femoral derotational osteotomies in cases where femoral anteversion exceeded 35° [10]. In another study by Huang et al., rotational deformities were assessed in skeletally immature patients with habitual patellar dislocation, revealing a mean femoral anteversion of 33°, which was comparable to recurrent patellar dislocation cases [13]. A recent case report by Ichikawa et al. described a 32‐year‐old patient with habitual patellar dislocation who had significant femoral anteversion (41.0°) and tibial torsion (40°). This patient underwent a double‐level derotational osteotomy with successful outcomes [14]. In our study of skeletally mature patients, we found that rotational deformities of the lower extremity in habitual patellar dislocation cases were significantly less severe compared with those in recurrent patellar dislocation. Although our findings do not definitively establish whether mild rotational deformity is a contributing factor to habitual patellar dislocation or a compensatory adaptation, it suggests that the association between habitual patellar dislocation and rotational deformity may not be as pronounced as observed in recurrent patellar dislocation cases.

Patella alta is widely recognised as a significant risk factor for recurrent patellar dislocation [7]. Conversely, the literature presents inconsistent findings regarding patellar height in cases of habitual patellar dislocation. Benoit et al. reported long‐term outcomes in eight children with habitual patellar dislocation, all of whom exhibited preoperative patella alta. This condition was corrected through distalization of the patella via complete mobilisation of the patellar tendon [3]. In another study by Niedzielski et al. [18], 11 paediatric patients with habitual patellar dislocation underwent extensive soft‐tissue procedures, revealing patella alta in five patients (45%) and patella baja in three (27%). Huang et al. compared radiological features between recurrent patellar dislocation and habitual patellar dislocation in children, noting a significantly lower Insall−Salvati index in the habitual patellar dislocation group [13]. Our current study focused exclusively on skeletally mature patients with habitual patellar dislocation. Interestingly, approximately half of these patients exhibited patella baja, with none showing patella alta—a contrast to findings in recurrent patellar dislocation. Unlike the aforementioned studies, which included paediatric patients, our study's inclusion criteria may explain these divergent results, as bony deformities may gradually relieve or further worsen with growth in habitual patellar dislocation. Therefore, in skeletally mature patients presenting with habitual patellar dislocation and concurrent patella baja, if the patella remains unreduced at full knee flexion following extensive soft tissue surgery, tibial tubercle proximalization could be considered. This approach not only aims to normalise patellar height but also plays a role in lengthening the extensor mechanism, thereby aiding patellar reduction in severe cases [9, 21, 22].

In cases of habitual patellar dislocation, the extensor mechanism often lacks sufficient length to allow full knee flexion with the patella properly centred. Consequently, the patella tends to track laterally along a shorter path of the extensor mechanism, permitting knee flexion only when the patella is laterally displaced. This can lead to some patients developing an ‘invisible patella’ on true lateral view at 30° of knee flexion due to severe contractures of the quadriceps mechanism. Huang et al. [13] found that 52.4% of patients with habitual patellar dislocation exhibited an ‘invisible patella’ at 30° of knee flexion, which aligns closely with our own findings (49.1%). The marked radiological distinction between knees with and without an ‘invisible patella’ underscores the potential impact of this unique feature on surgical decision‐making in skeletally mature patients with habitual patellar dislocation. First, knees with an ‘invisible patella’ demonstrated a significantly higher tibiofemoral rotation angle, a known indicator of the severity of patellar instability [16]. Therefore, the presence of an ‘invisible patella’ at 30° of knee flexion suggests that this subgroup of patients may present greater surgical challenges. Second, the TT‐TG distance was approximately 1.5 times greater in knees with an ‘invisible patella’ compared with those without, indicating a higher likelihood of requiring tibial tubercle medialization. This surgical intervention has previously been employed in the treatment of skeletally mature patients with habitual patellar dislocation [21].

The clinical relevance of this study lies in its detailed comparison of radiographic parameters between habitual patellar dislocation and recurrent patellar dislocation in skeletally mature patients. Understanding these differences is crucial for improving diagnostic accuracy and guiding surgical decisions. For instance, the study highlights that habitual patellar dislocation patients have a higher incidence of patella baja and less severe rotational deformities, suggesting that different surgical approaches, such as tibial tuberosity proximalization, may be necessary for habitual patellar dislocation.

There are several limitations to this study. First, the selection criteria may have introduced selection bias. Due to patient age restrictions, only patients older than 14 years received surgical treatment in our sports medicine department, and we did not include skeletally mature patients who were <14 years of age (albeit a small proportion), which limits the generalisability of our findings to all skeletally mature patients with habitual patellar dislocation. Second, this is a single‐centre cohort study in a tertiary referral centre which attracts many referrals outside its geographical catchment area. Therefore, the profile of our patients and clinical practices may not be typical of regional centres and the extrapolation of our findings to other populations can be questioned. Third, the goal of describing the anatomical features of habitual patellar dislocation in skeletally mature patients was accomplished, however, future studies should include patients of all ages to determine if these anatomical parameters change with age. Fourth, the CDI cannot be measured in approximately half of patients with habitual patellar dislocation, which prevents us from fully and accurately describing the patellar height in skeletally mature patients with habitual patellar dislocation. Another limitation is the lack of a control group from the general population.

## CONCLUSIONS

A distinct difference in bony anatomical features was observed between habitual and recurrent patellar dislocation in skeletally mature patients. Habitual patellar dislocation exhibited less severe rotational deformities of the lower extremity but showed poorer trochlear and patellar development, a larger TT‐TG distance and a higher incidence of patella baja compared with recurrent patellar dislocation.

## AUTHOR CONTRIBUTIONS

Zhijun Zhang participated in the study design, data collection and drafted the manuscript. Menglinqian Di and Tong Zheng carried out the radiological measurements. Daofeng Wang and Zheng Feng participated in the data collection and statistical analysis. Hui Zhang conceived of the study and participated in its design and helped to draft the manuscript. All authors read and approved the final manuscript.

## CONFLICT OF INTEREST STATEMENT

The authors declare no conflict of interest.

## ETHICS STATEMENT

All procedures performed in this retrospective study were in accordance with the ethical standards of the Beijing Jishuitan Hospital Capital Medical University, and this study was performed after obtaining approval from our institutional review board (IRB, 20231011).

## Data Availability

The data sets used or analysed during the current study are available from the corresponding author on reasonable request.
